# Complete Genome Sequence of *Pantoea ananatis* Strain NN08200, an Endophytic Bacterium Isolated from Sugarcane

**DOI:** 10.1007/s00284-020-01972-x

**Published:** 2020-04-03

**Authors:** Quan Zeng, GuoYing Shi, ZeMei Nong, XueLian Ye, ChunJin Hu

**Affiliations:** Microbiology Research Institute, Guangxi Academy of Agricultural Sciences, Nanning, 530007 People’s Republic of China

## Abstract

Stain NN08200 was isolated from the surface-sterilized stem of sugarcane grown in Guangxi province of China. The stain was Gram-negative, facultative anaerobic, non-spore-forming bacteria. The complete genome SNP-based phylogenetic analysis indicate that NN08200 is a member of the genus *Pantoea ananatis*. Here, we summarize the features of strain NN08200 and describe its complete genome. The genome contains a chromosome and two plasmids, in total 5,176,640 nucleotides with 54.76% GC content. The chromosome genome contains 4598 protein-coding genes, and 135 ncRNA genes, including 22 rRNA genes, 78 tRNA genes and 35 sRNA genes, the plasmid 1 contains 149 protein-coding genes and the plasmid 2 contains 308 protein-coding genes. We identified 130 tandem repeats, 101 transposon genes, and 16 predicted genomic islands on the chromosome. We found an indole pyruvate decarboxylase encoding gene which involved in the biosynthesis of the plant hormone indole-3-acetic acid, it may explain the reason why NN08200 stain have growth-promoting effects on sugarcane. Considering the pathogenic potential and its versatility of the species of the genus *Pantoea*, the genome information of the strain NN08200 give us a chance to determine the genetic background of interactions between endophytic enterobacteria and plants.

## Introduction

The genus *Pantoea* comprises several species that are associated with plants have been found, either as pathogenic or beneficial bacteria to plants [1, 2]. Some of the first identified members of *Pantoea* were plant pathogens, but many studies subsequently indicated that *Pantoea* exist in a multitude of environments and most of them do beneficial to bioremediation and plant growth [3–5]. There are many *Pantoea* strains isolated from plants, soil and environment and are currently being explored for agricultural applications [6, 7]. Approximately, 20 *Pantoea* species have been identified, having diverse characteristics [8]. The ubiquity, versatility and genetic tractability of *Pantoea* make it ideal for exploring niche specific adaptation and opportunism, and for the development of agricultural and environmental products [9, 10].

To obtain endophytes that have growth-promoting effects on host sugarcane plants and have potential for agricultural application, we attempted to isolate and identify endophytic bacteria associated with sugarcane plants grown in Guangxi Province, the major sugarcane and sugar-producing area of China. Bacterial strain NN08200 was isolated from surface-sterilized stems of a ROC22 sugarcane plant grown in Nanning, Guangxi, China. We had determined the plant growth-promoting potential of strain NN08200 to sugarcane under a greenhouse condition [11]. Moreover, we observed the strain NN08200 colonization at the roots and aerial parts of micropropagated sugarcane plantlets with fluorescence microscopy and confocal microscopy. Sequence determinations and phylogenetic analysis of the 16S rRNA gene indicated that strain NN08200 is affiliated with the genus *Pantoea*, and the strain was preserved in the China General Microbiological Culture Collection Center, with the preservation number CGMCC No. 5438. Here, we present a summary of the features of strain NN08200 and its complete genome sequence, which provides a reference for resolving the phylogeny and taxonomy of closely related strains and genetic information to study the plant growth-promoting potential and plant-associated lifestyle of strain NN08200.

## Organism Information

### Classification and General Features

**Fig. 1:**
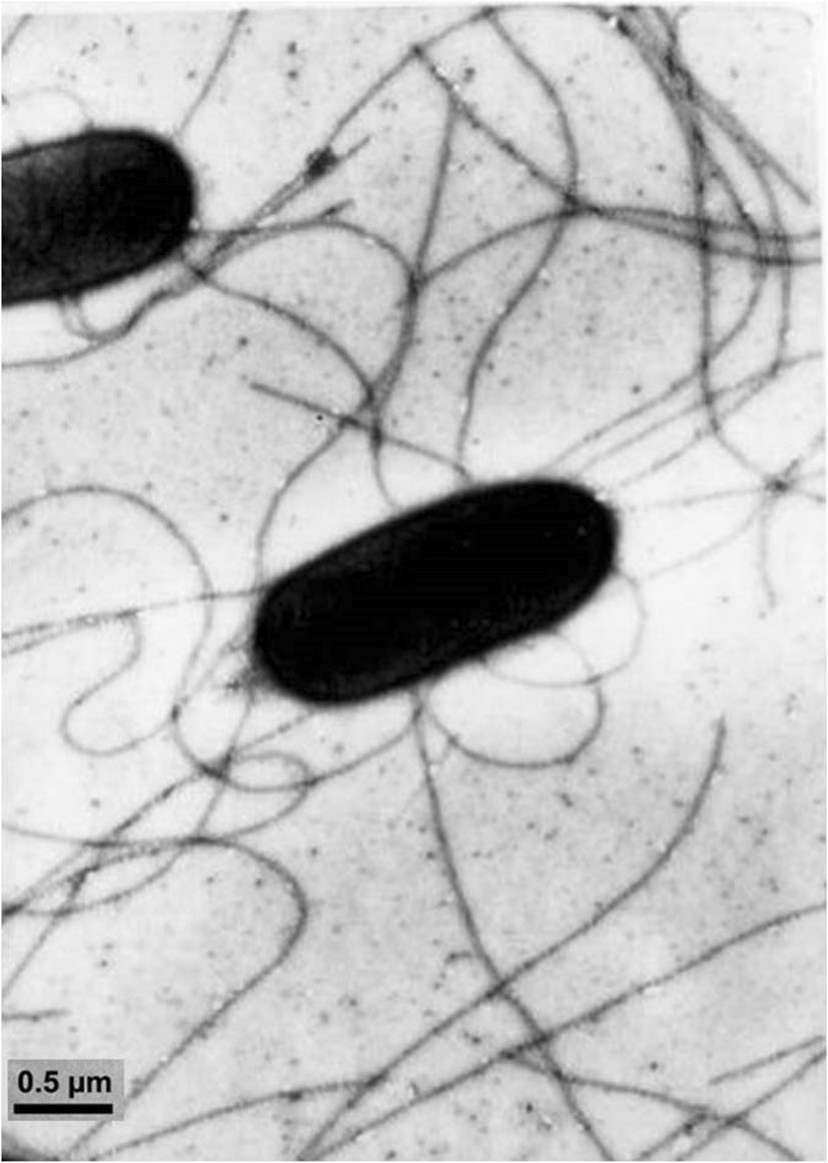
Transmission electron microphotograph of the *Pantoea ananatis* strain NN08200

Strain NN08200 is a Gram-negative, non-spore-forming, motile rod with peritrichous flagella (Fig. 1). This bacterium was able to grow in anaerobic using cooked meat medium with thermal melting vaseline and aerobic using beef extract medium, and grew optimally between 28 and 32 °C (Table 1). It forms circular, convex, smooth colonies on nutrient agar; in addition, it grows well on Ashby nitrogen-free culture medium, showing round, transparent colonies. Strain NN08200 is a species of *Pantoea*, showing several differences from the *Pantoea* species described so far. The strain is an endophyte from sugarcane. It is positive for indole production, nitrate reduction and arginine decarboxylase and lysine decarboxylase activity.

**Table 1:** Classification and general features of *Pantoea ananatis* strain NN08200

MIGS ID	Property	Term	Evidence code
	Current	Domain *Bacteria*	TAS [12]
	Classification	Phylum *Proteobacteria*	TAS [13]
		Class *Gammaproteobacteria*	TAS [14–17]
		Order *Enterobacterales*	TAS [18]
		Family *Erwiniaceae*	TAS [19]
		Genus *Pantoea*	TAS [11]
		Species *Pantoea ananatis*	TAS [11]
		Strain: NN08200	TAS [11]
	Gram strain	Negative	TAS [11]
	Cell shape	Rod	TAS [11]
	Motility	Motile	TAS [11]
	Sporulation	Non-sporulating	TAS [11]
	Temperature range	Mesophile	TAS [11]
	Optimum temperature	28–30℃	IDA
	Carbon source	Sucrose, flucose, fructose, galactose, maltose	IDA
	Energy source	Chemoorganotroph	IDA
MIGS-6	Habitat	Soil, plants	TAS [11]
MIGS-6.3	Salinity	0–4% NaCl	IDA
MIGS-22	Oxygen	Anaerobic and aerobic	TAS [11]
MIGS-23	Isolation	Stem of sugarcane cultivar GT22	TAS [11]
MIGS-15	Biotic relationship	Free-living, endophytic	TAS [11]
MIGS-14	Pathogenicity	No reported	
MIGS-4	Geographic location	Nanning, Guangxi, China	TAS [11]
MIGS-5	Sample collection time	2008	TAS [11]
MIGS-4.1	Longitude	107.37	NAS
MIGS-4.2	Latitude	22.40	NAS
MIGS-4.3	Depth	0.3–0.5 m above the surface	IDA
MIGS-4.4	Altitude	123 m	NAS

Evidence codes: IDA: Inferred from Direct Assay; TAS: Traceable Author Statement (i.e., a direct report exists in the literature); NAS: Non-traceable Author Statement (i.e., not directly observed for the living, isolated sample, but based on a generally accepted property for the species, or anecdotal evidence)

**Fig. 2:**
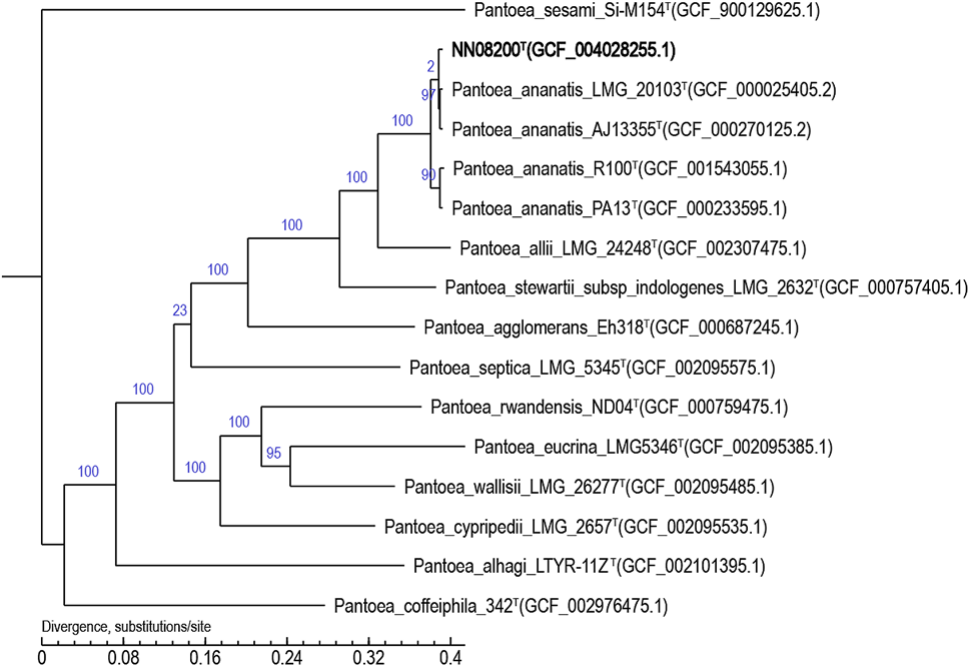
Phylogenetic tree based on the genome sequences showing the phylogenetic position of strain NN08200 and other strains belonging to the genus *Pantoea*. A PHYML method was beedn used to build the phylogenetic tree based on SNP of complete genomes for strain belonging to the genus Pantoea constructed by TreeBeST

A PHYML method phylogenetic tree based on SNP of complete genomes for strain belonging to the genus Pantoea constructed by TreeBeST (Fig. 2) showed that strain NN08200 is most closely related to strains belonging to the *Pantoea ananatis *[20]. Genomes gene sequences from the following strains were used to construct the phylogenetic tree: *P. sesame* Si-M154, taxonomy ID: 1881110; *P. ananatis* LMG 20103, taxonomy ID: 706191; *P. ananatis* AJ13355, taxonomy ID: 932677; *P. ananatis* R100, taxonomy ID:; *P. ananatis* PA13, taxonomy ID: 1095774; *P. allii* LMG_24248, taxonomy ID: 574096; *P. stewartii subsp_indologenes* LMG 2632, taxonomy ID: 66270; *P. agglomerans* Eh318, taxonomy ID: 1408177; *P. septica* LMG 5345, taxonomy ID: 472695; *P. rwandensis* ND04, taxonomy ID: 1076550; *P. eucrina* LMG 5346, taxonomy ID: 472693; *P. wallisii* LMG 26277, taxonomy ID: 1076551; *P. cypripedii* LMG 2657, taxonomy ID: 55209; *P. alhagi* LTYR-11Z, taxonomy ID: 1891675; *1* 342, taxonomy ID: 1465635.

### Genome Sequencing Information Genome Project History

*Pantoea ananatis* strain NN08200 was selected for sequencing based on its taxonomic significance and because it could be used in promoting plant growth. The genome sequence is deposited in GenBank with the accession number CP035034. Information about the genome sequencing and its association with MIGS version 2.0 compliance is shown in Table 2.

**Table 2:** Genome sequencing project information for *Pantoea ananatis* NN08200

MIGS ID	Property	Term
MIGS-31	Finishing quality	Finished
MIGS-28	Libraries used	10 kb SMRT Bell library
MIGS-29	Sequencing platforms	PacBio RS II
MIGS-31.2	Fold coverage	166×
MIGS-30	Assemblers	SMRT portal
MIGS-32	Gene calling method	GeneMarkS
	Genome database release	Genbank
	Genbank ID	CP035034
	Genbank date of release	Jan 17,2019
MIGS-13	Source material identifier	NN08200
	Project relevance	Taxonomy, biotechnology

### Growth Conditions and DNA Isolation

*P. ananatis* strain NN08200 was grown in liquid Luria–Bertani medium at 28 °C until stationary phase. Genomic DNA was extracted using a TIANamp bacterial DNA kit (Tiangen Biotech, Beijing, China). The quantity and quality of DNA were assessed using a NanoDrop spectrophotometer (Thermo Scientific, USA).

### Genome Sequencing and Assembly

The genomic DNA of *P. ananatis* strain NN08200 was first constructed into a 10-kb SMRT Bell library and sequenced using the PacBio RS II sequencing system. Low-quality reads were filtered by the SMRT portal (version 2.3.0) and the filtered reads were assembled to generate five contigs containing 5,176,640 bases [21, 22]. The final assembly of the genome provided an average of 166-fold coverage. The five contigs were scaffolding to three circular sequences. The fully assembled *P. ananatis* strain NN08200 genome is composed of a 4.7-M base pair chromosome, and two plasmids, whose sizes were 125k and 307k base pairs, respectively.

### Genome Annotation

The complete sequence of *P. ananatis* strain NN08200 was analyzed using GeneMarkS (version 4.17) to retrieve protein coding genes [23]. Transfer RNA (tRNA) genes were predicted by tRNAscan-SE [24]. Ribosomal RNA (rRNA) genes were analyzed by rRNAmmer [25]. Transposon PSI was used to predict transposons based on the homologous blast method. RepeatMasker (version open-4.0.5) and TRF (tandem repeats finder, version 4.07b) were used for identification of interspersed nuclear elements and tandem repeats, respectively [26, 27]. SlandPath-DIOMB (version 0.2) was used for identification of genomic islands [28].

### Genome Properties

**Fig. 3:**
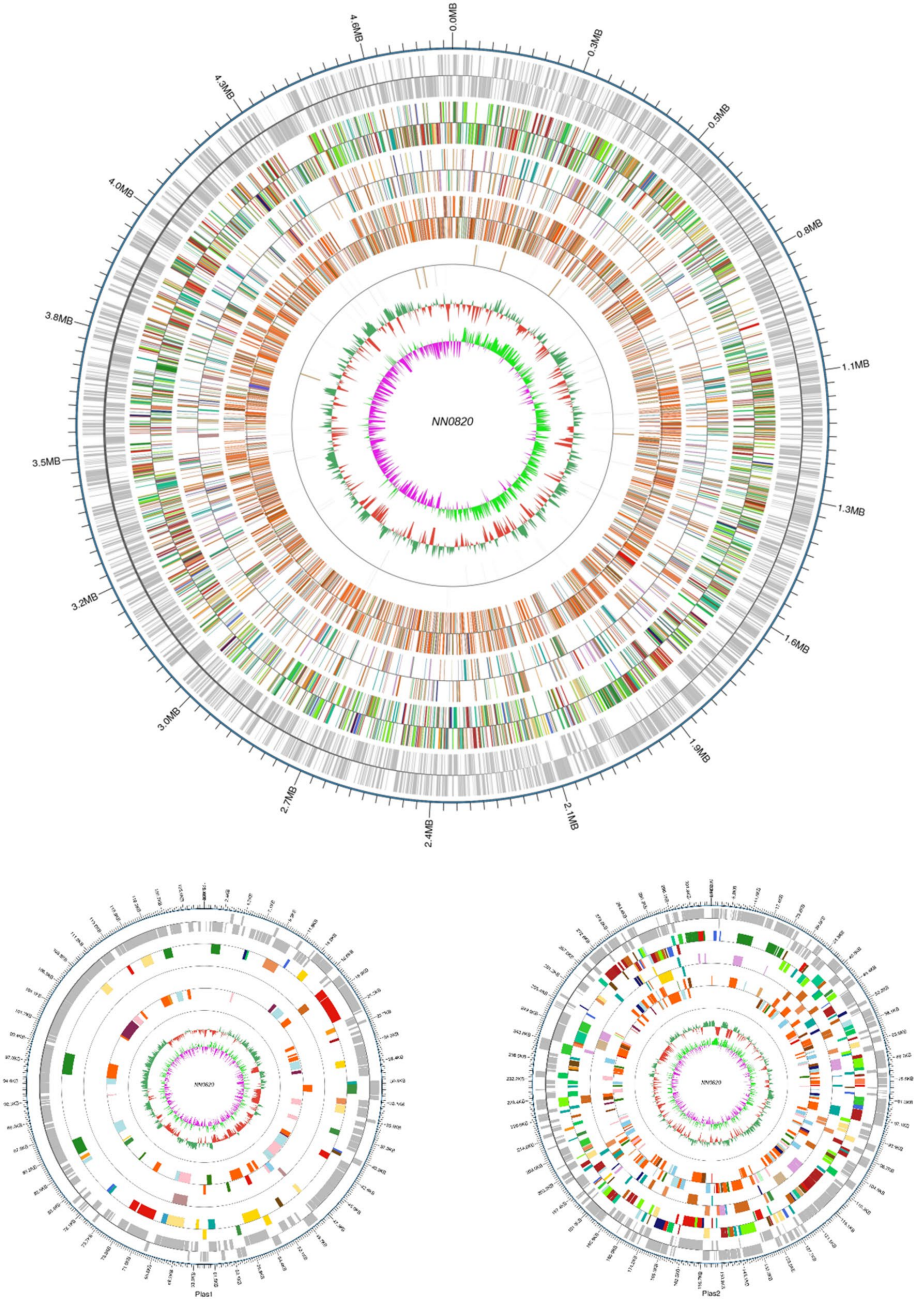
Graphical circular map of the chromosome and plasmids of Pantoea ananatis NN08200 by Circos. From outside to the center: Coding genes on forward and reverse strands, the results of gene function annotation (including genes, COG, KEGG, GO), ncRNAs

The genome of strain NN08200 contains a single chromosome of 4,743,568 nucleotides with 53.8% G+C content and two plasmids, one of 125,402 nucleotides with 56.47% G+C content and another of 307,670 nucleotides with 52.17% G+C content. The chromosome contains 4733 predicted genes: 4598 protein-coding genes and 135 RNA genes including 78 tRNA genes, 35 sRNA genes, and 22 rRNA genes (Table 3; Fig. 3). The plasmid 1 contains 149 protein-coding genes and the plasmid 2 contains 308 protein-coding genes. Ciros was used to show the genome and the result of gene function annotation [29]. In total, 4369 genes were assigned in Clusters of Orthologous Groups of proteins (COG) functional categories and they are listed in Table 4.

**Table 3:** Nucleotide content and gene count levels of the *P. ananatis* NN08200 genome

Attribute	Value	% of total
Size (bp)	5,176,640	100.00
G+C content (bp)	2,834,728	54.76
Coding region (bp)	4,480,173	86.55
Total genes	4733	100.00
RNA genes	135	2.85
Protein-coding genes	4598	97.15
Genes assigned to COGs	4369	86.43
Genes with signal peptides	412	8.96
Genes with transmembrane helices	341	7.42
Chromosome size (bp)	4,743,568	91.6%
Chromosome G+C content (bp)	2,552,158	53.8%
Plasmid 1 size (bp)	125,402	2.4%
Plasmid 1 G+C content (bp)	70,096	56.47%
Plasmid 2 size (bp)	307,670	7.1%
Plasmid 2 G+C (bp)	160,525	52.17%

**Table 4:** Number of genes associated with the 25 general COG functional categories

Code	Value	% of total	Description
A	1	0.02	RNA processing and modification
C	191	3.78	Energy production and conversion
D	52	1.03	Cell cycle control, cell division, chromosome partitioning
E	418	8.27	Amino acid transport and metabolism
F	105	2.08	Nucleotide transport and metabolism
G	451	8.92	Carbohydrate transport and metabolism
H	200	3.96	Coenzyme transport and metabolism
I	150	2.97	Lipid transport and metabolism
J	269	5.32	Translation, ribosomal structure and biogenesis
K	351	6.94	Transcription
L	162	3.2	Replication, recombination and repair
M	287	5.68	Cell wall/membrane/envelope biogenesis
N	133	2.63	Cell motility
O	146	2.89	Posttranslational modification, protein turnover, chaperones
P	256	5.06	Inorganic ion transport and metabolism
Q	89	1.76	Secondary metabolites biosynthesis, transport and catabolism
R	352	6.96	General function prediction only
S	226	4.47	Function unknown
T	256	5.06	Signal transduction mechanisms
U	98	1.94	Intracellular trafficking, secretion, and vesicular transport
V	90	1.78	Defense mechanisms
W	30	0.59	Extracellular structures
X	56	1.1	Mobilome: prophages, transposons
–	686	13.57	Not in COGs

### Insights from the Genome

Here we present the complete genome sequence of *Pantoea ananatis* strain NN08200. Protein-coding sequences accounted for 4598(97.15%) of the total of 4733 genes identified. 54 complete genomes of P. ananatis have been download from NCBI to performed an Average Nucleotide Identity (ANI) analysis with strain NN08200 [30]. The results justified the conclusion of phylogenetic analysis, strain NN08200 with other strains resulted in a high ANI (> 95%). The results suggested that the strain NNo8200 belongs to the P. ananatis. NN08200 and P. ananatis S8 resulted in the highest ANI (99.2%) and show that they are similar than other strains.

## Conclusion

In this study, we present the complete genome sequence of *Pantoea ananatis* strain NN08200, an endophyte from sugarcane. The genome of *P. ananatis* NN08200 consists of a 4,743,568-bp long chromosome, containing 4598 protein coding genes. *P. ananatis* NN08200 also contains two plasmids. To analyze the complete genome sequence of *Pantoea ananatis* strain NN08200, we found an indole pyruvate decarboxylase encoding gene which involved in the biosynthesis of the plant hormone indole-3-acetic acid [31], it may promote plant growth by improving the synthesis of indoleacetic acid. The new genomic data will facilitate future applications of this strain in agricultural production.

## Data Availability

The datasets generated and analysed during the current study are available in the NCBI repository, (www.ncbi.nlm.nih.gov/bioproject/PRJNA514184).
